# Juvenile polyposis syndrome might be misdiagnosed as familial adenomatous polyposis: a case report and literature review

**DOI:** 10.1186/s12876-020-01238-7

**Published:** 2020-06-01

**Authors:** Xian Hua Gao, Juan Li, Zi Ye Zhao, Xiao Dong Xu, Yi Qi Du, Hong Li Yan, Lian Jie Liu, Chen Guang Bai, Wei Zhang

**Affiliations:** 1 Department of Colorectal Surgery, Changhai Hospital, 168 Changhai Road, Shanghai, 200433 China; 2 Hereditary Colorectal Cancer Center and Genetic Block Center of Familial Cancer, Changhai Hospital, Shanghai, China; 3 Department of Nephrology, Changhai Hospital, Shanghai, China; 4 Department of Gastroenterology, Changhai Hospital, Shanghai, China; 5 Reproductive Medicine Center, Changhai Hospital, Shanghai, China; 6 Department of Pathology, Changhai Hospital, 168 Changhai Road, Shanghai, 200433 China

**Keywords:** Juvenile polyposis syndrome, Familial adenomatous polyposis, Misdiagnosis, Dysplasia, Adenoma

## Abstract

**Background:**

Juvenile polyposis syndrome (JPS) is a rare disorder characterized by the presence of multiple juvenile polyps in the gastrointestinal tract, and germline mutations in SMAD4 or BMPR1A. Due to its rarity and complex clinical manifestation, misdiagnosis often occurs in clinical practice.

**Case presentation:**

A 42-year-old man with multiple pedunculated colorectal polyps and concomitant rectal adenocarcinoma was admitted to our hospital. His mother had died of colon cancer. He was diagnosed with familial adenomatous polyposis (FAP) and underwent total proctocolectomy and ileal pouch anal anastomosis. Two polyps were selected for pathological examination. One polyp had cystically dilated glands with slight dysplasia. The other polyp displayed severe dysplasia and was diagnosed as adenoma. Three years later, his 21-year-old son underwent a colonoscopy that revealed more than 50 pedunculated colorectal juvenile polyps. Both patients harbored a germline pathogenic mutation in *BMPR1A*. Endoscopic resection of all polyps was attempted but failed. Finally, the son received endoscopic resection of polyps in the rectum and sigmoid colon, and laparoscopic subtotal colectomy. Ten polyps were selected for pathological examination. All were revealed to be typical juvenile polyps, with cystically dilated glands filled with mucus. Thus, the diagnosis of JPS was confirmed in the son. A review of the literatures revealed that patients with JPS can sometimes have adenomatous change. Most polyps in patients with JPS are benign hamartomatous polyps with no dysplasia. A review of 767 colorectal JPS polyps demonstrated that 8.5% of the polyps contained mild to moderate dysplasia, and only 0.3% had severe dysplasia or cancer. It is difficult to differentiate juvenile polyps with dysplasia from adenoma, which could explain why juvenile polyps have been reported to have adenomatous changes in patients with JPS. Therefore, patients with JPS, especially those with concomitant dysplasia and adenocarcinoma, might be easily diagnosed as FAP in clinical practice.

**Conclusions:**

Juvenile polyp with dysplasia is often diagnosed as adenoma, which might lead to the misdiagnosis of JPS as FAP. The differential diagnosis of JPS versus FAP, should be based on comprehensive evaluation of clinical presentation, endoscopic appearance and genetic investigations; not on the presence or absence of adenoma.

## Background

Juvenile polyposis syndrome (JPS) was first described in 1964 [1]. It is a rare (approximately one in every 100,000 individuals) autosomal dominant disease that is characterized by the occurrence of several juvenile polyps in the gastrointestinal tract [2, 3]. Juvenile polyp is a specific type of hamartomatous polyp. The term “juvenile” refers to the polyp histology rather than the age of onset of the polyp [3]. An isolated juvenile polyp is not diagnostic of JPS and occurs in approximately 2% of children and adolescents [3]. In contrast to Peutz-Jeghers syndrome (PJS), in which small bowel polyps are common, patients with JPS develop polyps primarily in the stomach and colon [2, 4]. JPS is associated with an increased risk of gastrointestinal cancer. The cumulative risks of colorectal and gastric cancers are estimated to be approximately 40–50 and 20%, respectively [2–5]. Pancreatic cancer and duodenal cancer have also been reported [4–6]. Malignant tumor has not been found in other parts of the small intestinal bowel.

Germline mutations in SMAD family member 4 (SMAD4) or bone morphogenetic protein receptor type 1A (BMPR1A), have been found in 50–60% of patients with JPS [4, 7–9]. Both genes are involved in the bone morphogenetic protein (*BMP*) – transforming growth factor-beta (*TGF-β*) signaling pathway [5]. Most of the variants are point mutations or small base pair deletions in the coding regions, and approximately 15% of the variants are large deletions or recombinations. Mutations in *ENG* related with the *TGF-β* signaling pathway have also been reported in some patients [2, 6]. However, in a large proportion of JPS cases, the responsible gene mutation was not identified [2]. Approximately 20–50% of JPS cases have no family history and they may harbor de novo mutations [3, 4, 6].

JPS often presents in childhood before puberty. Typical symptoms include hematochezia, anemia, diarrhea, abdominal pain, intussusception, intestinal obstruction, and polyp prolapse [2–6]. Patients with JPS with *SMAD4* mutations can have markedly higher risks of gastric polyps and gastric cancer than patients with the *BMPR1A* mutation [3, 5, 10, 11]. Most patients with JPS who harbor *SMAD4* variants might have concomitant hereditary hemorrhagic telangiectasia (HHT) or Rendu-Osler-Weber syndrome [2–4], which is characterized by vascular malformations with epistaxis, mucocutaneous telangiectasia, and arteriovenous malformations of the lungs, liver, and brain [5, 12, 13]. Cardiac atrial and ventricular septal defects and aneurysms of the central nervous system and pulmonary circulation have also been reported in JPS cases [2].

Due to its rarity and complex clinical manifestation, misdiagnosis of JPS may occur in clinical practice. Here, we report a father-son case of JPS misdiagnosed as familial adenomatous polyposis (FAP). The literature was reviewed to elucidate the possible reason for the misdiagnosis and to determine how a more accurate diagnosis can be made in the future.

## Case presentation

### Clinical manifestation and treatment process of the father

**Fig. 1:**
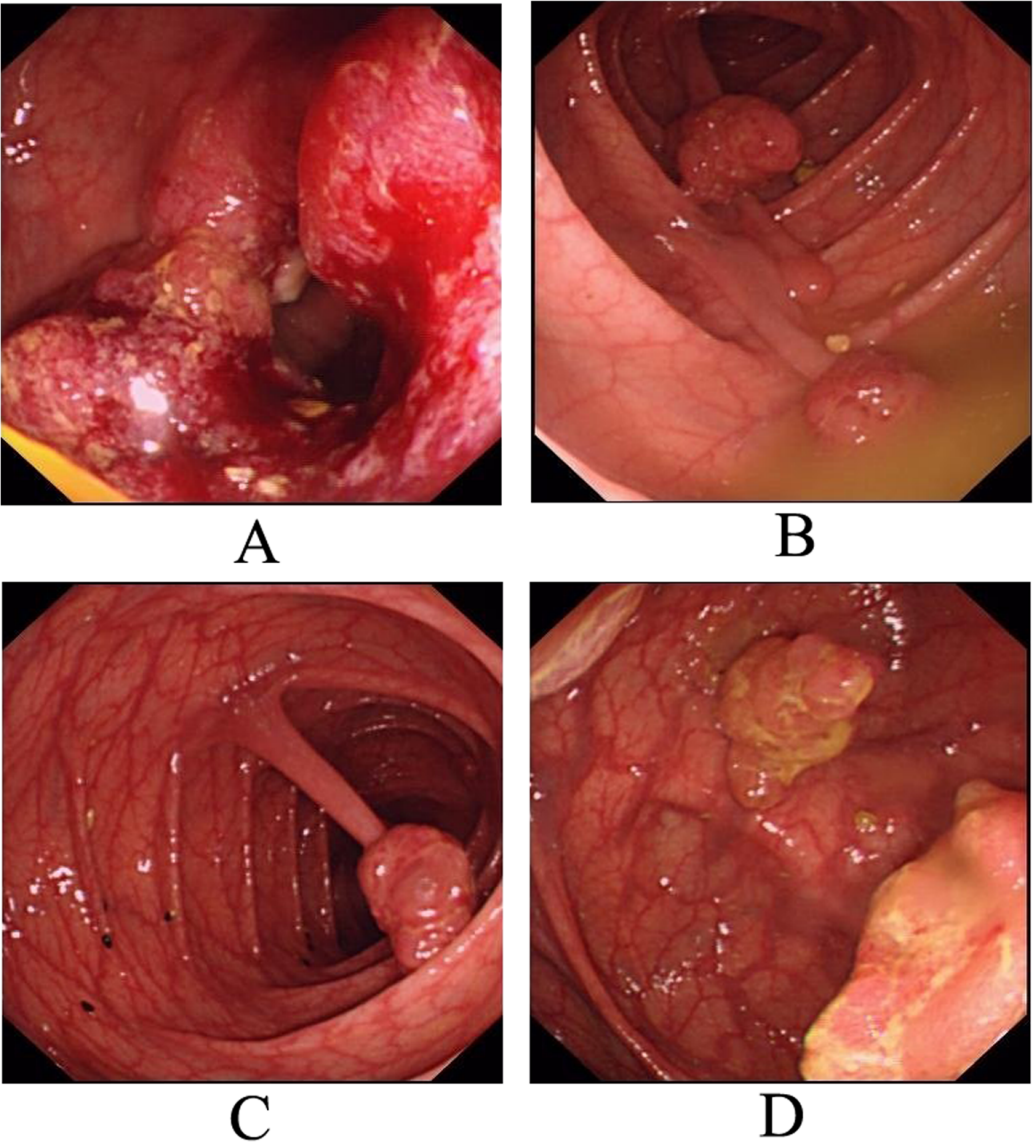
Colonoscopic findings of the father. **a**: rectal adenocarcinoma; **b**, **c**: pedunculated polyps; **d**: sessile polyps with mulberry shape

**Fig. 2:**
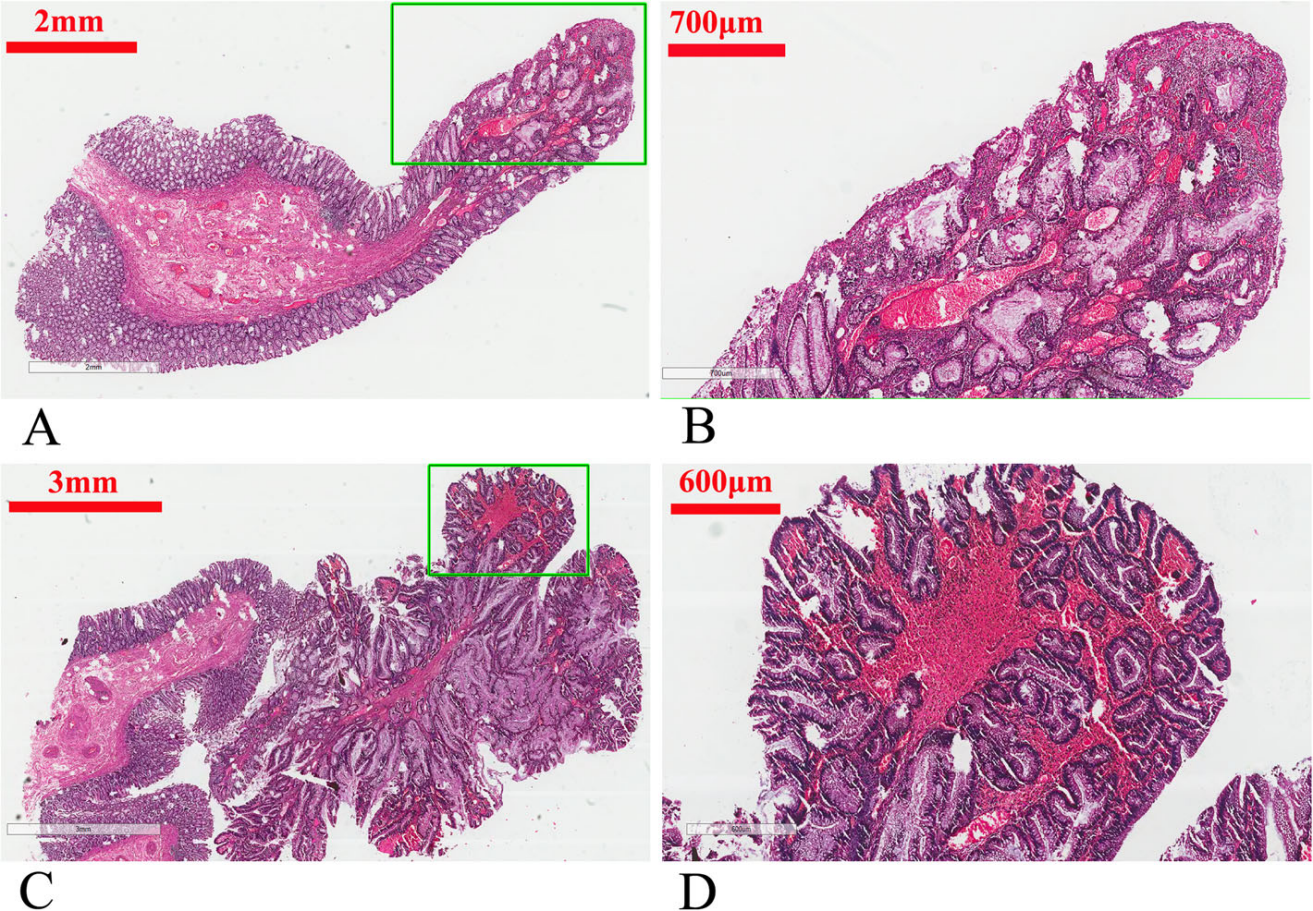
Pathological manifestation of the two examined colorectal polyps in the father. **a** Gross appearance of one polyp. The right half is the polyp, and the left half is normal mucosa. **b** Magnified image of the selected rectangular area from panel A, showing cystically dilated glands with slight dysplasia. **c** Gross appearance of the other polyp. The right half was the polyp, and the left half was normal mucosa. **d** Magnified image of the selected rectangular area from panel **c**, showing glands with severe dysplasia. It was diagnosed as adenoma

A 42-year-old Chinese man with colorectal polyps and concomitant rectal cancer was admitted to our department. His mother had died of colorectal polyposis and colon cancer at 53 years old. Digital rectal examination showed a circular mass located 4 cm above the anal verge. No other abnormality was observed by physical examination. Laboratory data also revealed no abnormalities. Colonoscopy revealed an ulcerative mass occupying a circle of the intestine in the rectum, located 4 cm from the anal verge. Multiple polyps (40–50, diameter 0.5–3 cm) were also observed throughout the colorectum, and some were pedunculated polyps (Fig. 1). Pathological examination of a biopsy sample identified the rectal mass as adenocarcinoma (Fig. 2). Following a diagnosis of FAP, total proctocolectomy (TPC) and ileal pouch anal anastomosis (IPAA) was performed. The rectal mass was revealed to be an adenocarcinoma. Only two colorectal polyps were selected from the resected colon for pathological examination. One polyp had cystically dilated glands with slight dysplasia. The other polyp displayed severe dysplasia and was diagnosed as adenoma. Recovery was uneventful and the ileostoma was closed 3 month later. After ileostoma closure, he experienced severe fecal urgency, with a daily frequency of more than 20 times even after medication with loperamide. Multiple lung metastases were detected one year after the initial surgery.

### Clinical manifestation and treatment process of the son

**Fig. 3:**
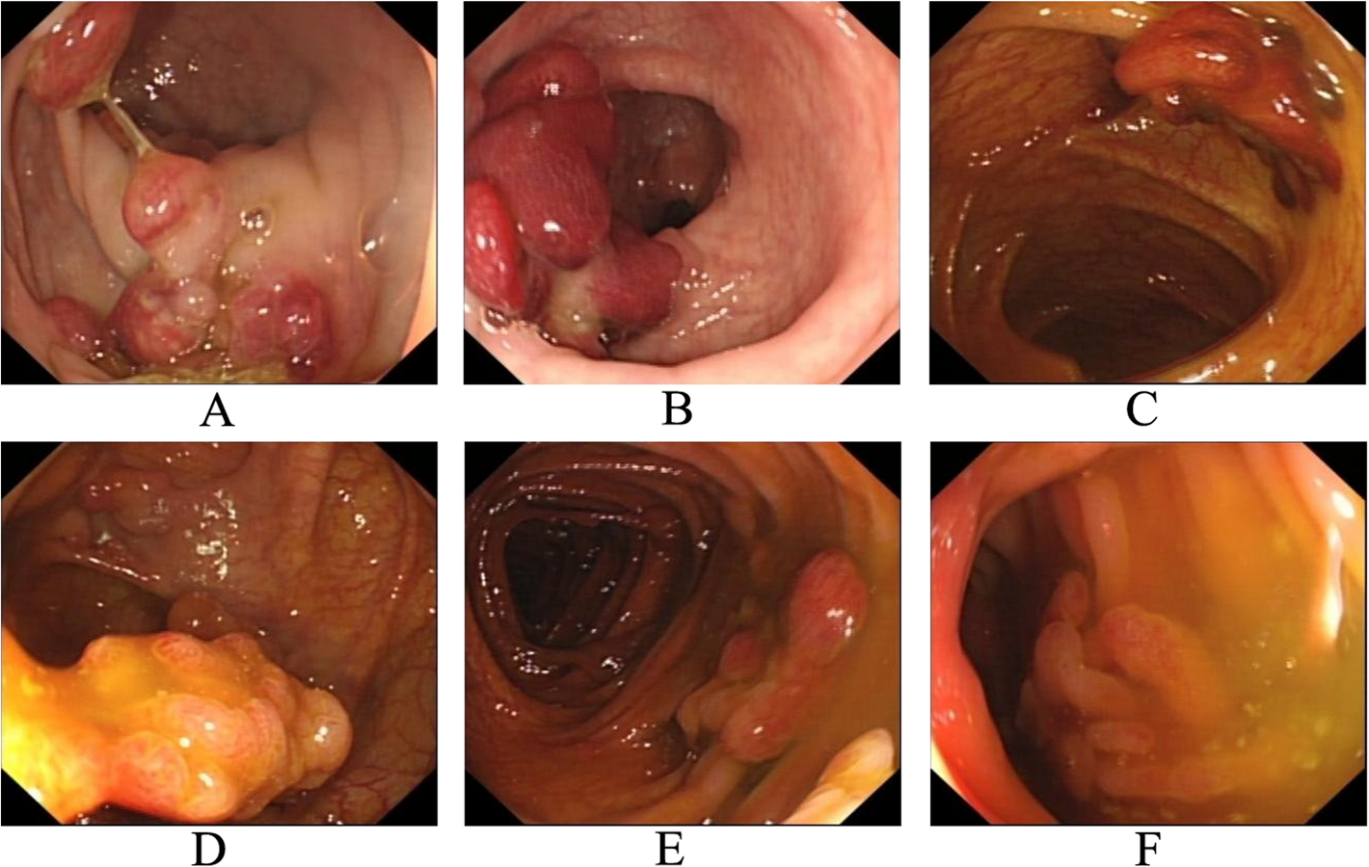
Colonoscopic manifestations of the son. **a-f** multiple polypoid lesions in the rectum and colon

**Fig. 4:**
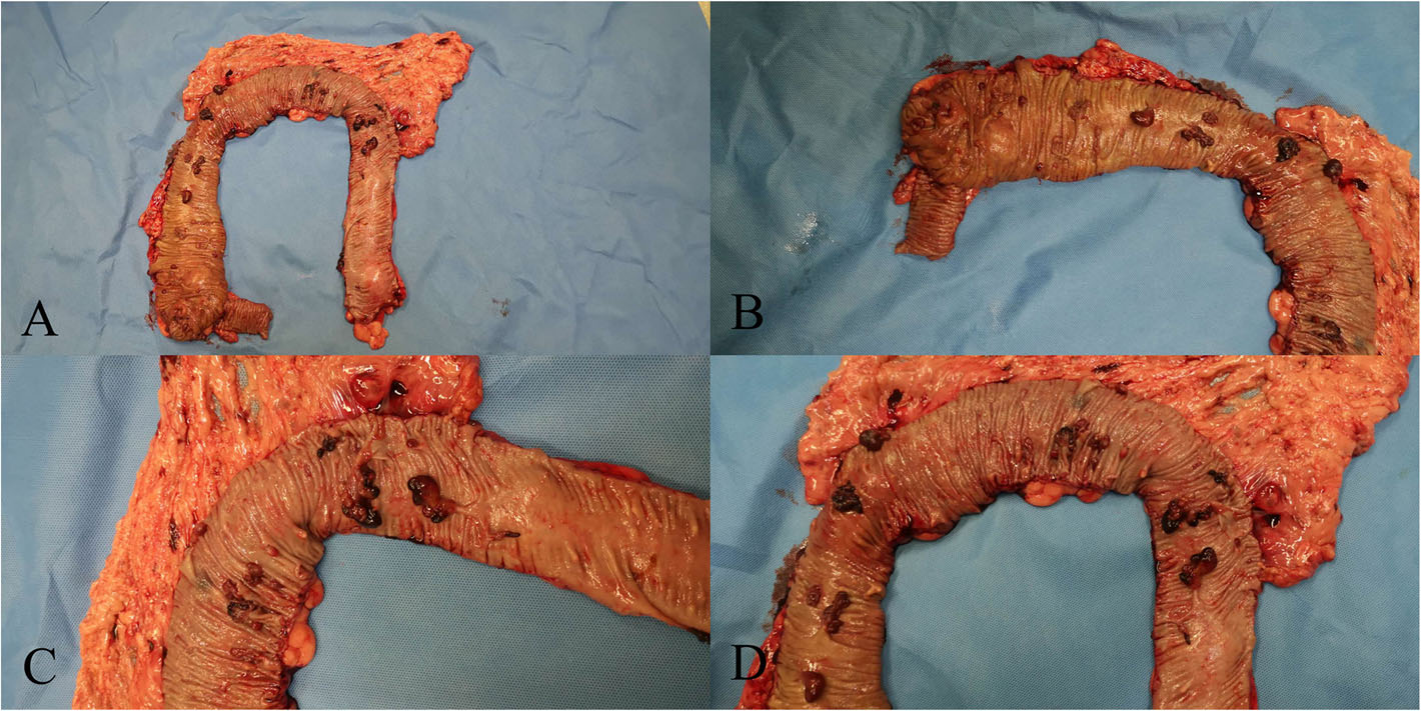
Resected specimen of the subtotal colon from the son. **a** Whole view of the resected specimen, from the cecum to the descending codlon. **b** Polyps in the cecum and ascending colon. **c** Polyps in the hepatic flexure colon. **d** Huge polyps in the hepatic flexure, transverse colon, and splenic flexure

**Fig. 5:**
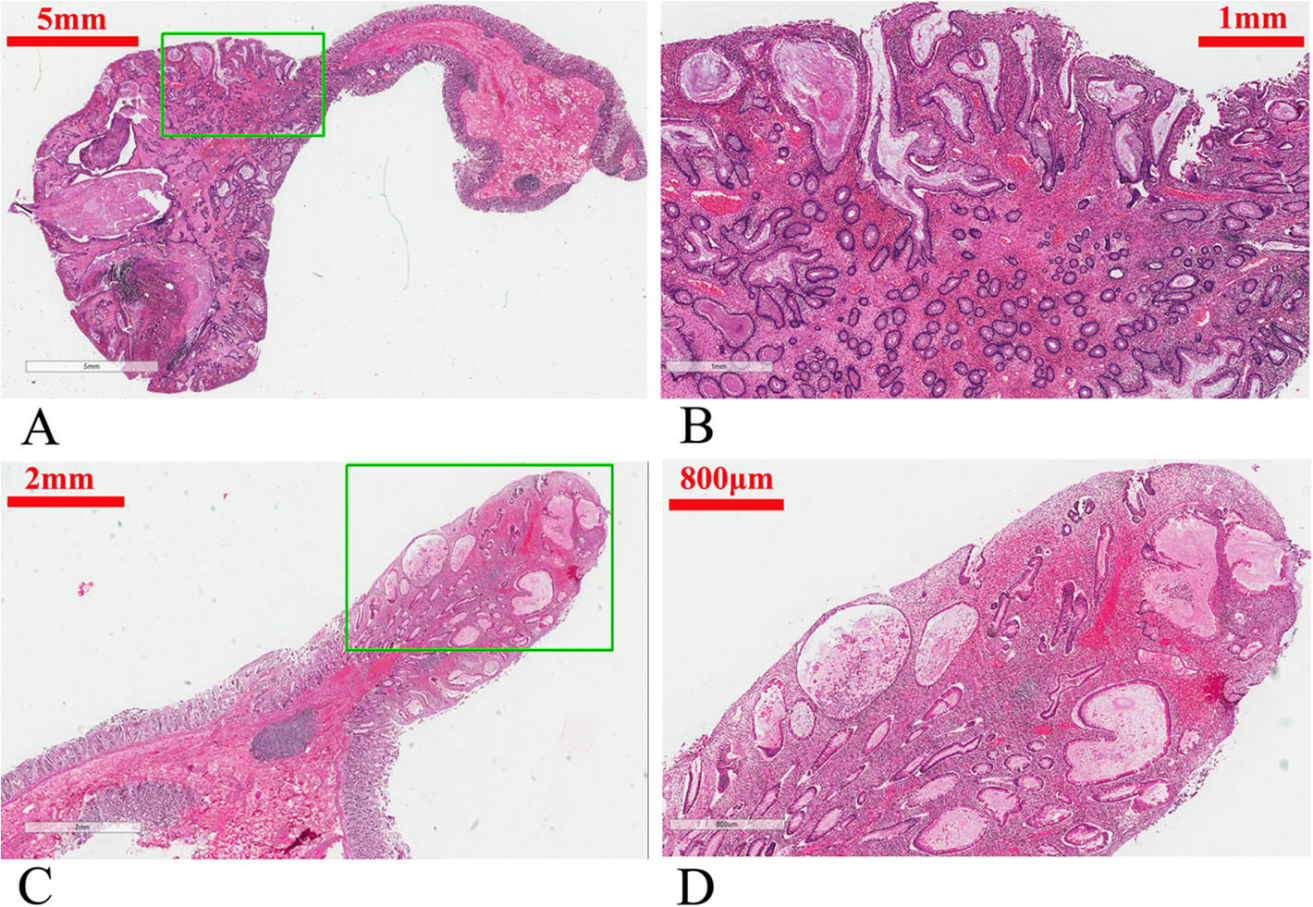
Pathological manifestation of two examined colorectal polyps in the son. **a** Gross appearance of one polyp. The left half was the polyp, and the right half was normal mucosa. **b** Magnified image of the selected rectangular area from panel **a**, showing typical juvenile polyp, with cystically dilated glands, abundant mucus in the gland cavity, cuboidal epithelium, abundance of edematous lamina propria. **c** Gross appearance of the other polyp. The right half was the polyp, and the left half was normal mucosa. **d**: Magnified image of the selected rectangular area from panel **c**, showing typical juvenile polyp

After the patient was misdiagnosed as FAP, a colonoscopy was suggested for his 21-year-old son. The colonoscopy performed at our hospital revealed more than 50 polyps measuring 0.5–2.5 cm in diameter in the colon and rectum (Fig. 3). Esophagogastroduodenoscopy (EGD) revealed no polyps in the stomach. He was also suggested to undergo TPC + IPAA. He declined and came to our hereditary colorectal cancer counseling clinic. Gene mutation testing indicated that both he and his father had a germline mutation in *BMPR1A* (c.949_952delCTCT). This variant was classified as pathogenic. Finally, the son and his father were diagnosed with JPS. Endoscopic resection of all polyps was attempted for the son, but failed because of the presence of many huge polyps. After careful discussion with his family, an individual plan was designed for him, which involved endoscopic resection of polyps in the rectum and sigmoid colon, and laparoscopic subtotal colectomy. Most of the colon (from cecum to descending colon) was removed and the terminal ileum was connected with the sigmoid colon with a stapled side-to-end anastomosis. The resected colon specimen contained numerous pedunculated polyps in his colon (Fig. 4). Ten polyps were selected for pathological examination. All displayed cystically dilated glands filled with mucus lined by cuboidal or columnar epithelium, abundance of edematous lamina propria with inflammatory cells, and the absence of smooth muscle in the stroma. No dysplasia or cancer was evident in these polyps (Fig. 5). All examined polyps were proved to be typical juvenile polyps, so the diagnosis of JPS was confirmed in the son. He remains in excellent condition with normal sexual and urinary functions. His fecal frequency was once per day at one year after surgery.

## Mutation analysis

**Fig. 6:**
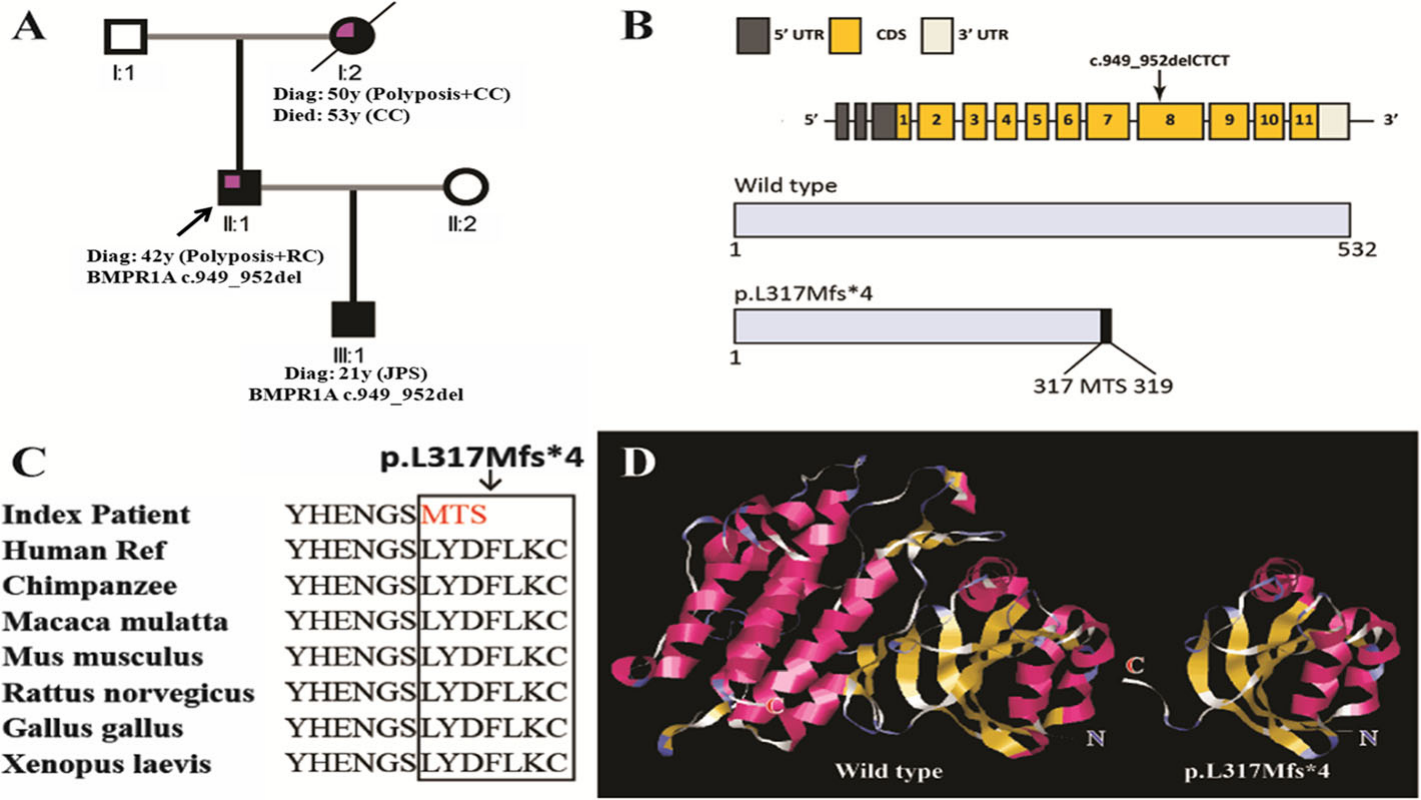
**a** Genogram of the proband (upper left quarter in purple color indicating colorectal cancer; black color indicating JPS; Diag: diagnosis; CC: colon cancer; RC: rectal cancer). **b** The structure of the *BMPR1A* gene. The novel mutation is in exon 8. **c** Evolutionary conservation of amino acid residues altered by *BMPR1A*: c.949_952delCTCT (p.L317Mfs) across different species. **d** Local structures around the mutation site of the wild type and mutant *BMPR1A* protein generated by Swiss-model online software. The structural differences are apparent

Written informed consent was obtained from the two patients for a 139-gene panel by next generation sequencing. The patients received standard genetic counseling in our hereditary colorectal cancer counseling clinic. Pedigree of family is presented in Fig.6a. Blood samples were collected and sent to a commercial company for analysis of genomic mutations of hereditary cancer genes. The genetic test was performed using a 139-gene panel from the GENETRON HEALTH (Beijing, China). All of these 139 genes are hereditary cancer related genes (Supplemental Table 1), including all known polyposis related genes, such as *SMAD4, BMPR1A, STK11, PTEN, APC, MUTYH, POLE, POLD1* and *NTHL1*.

Direct sequencing of the genomic DNA of the father and son showed that the *BMPR1A*: c.949_952delCTCT pathogenic variant was present in exon 8 of the *BMPR1A* gene (Fig.6b). This deletion is a frameshift variant predicted to lead to a substitution of a leucine with a methionine at codon 317 and a premature stop codon at position 320 (BMPR1A: p.L317Mfs*4). This variant had been reported in the ClinVar online database [14], and it is a truncating mutation. The mutation site is highly conserved among different species, which indicates that the site is vital for normal function (Fig.6c). The structure prediction by Swiss-model online software showed that there were apparent differences between the wild and mutant protein (Fig.6d). It was classified as a pathogenic variant according to the American College of Medical Genetics and Genomics criteria [15]. No *SMAD4* gene mutations were detected in the patients’ genomic DNA.

## Discussion and conclusions

Juvenile polyps are histologically characterized by cystically dilated glands filled with mucus lined by cuboidal or columnar epithelium, abundance of edematous lamina propria with inflammatory cells, and absence of smooth muscle in the stroma [5, 11]. The diagnosis of JPS can be confirmed if any one of the following criteria are met: (1) five or more juvenile polyps of the colorectum, (2) multiple juvenile polyps throughout the gastrointestinal tract, and (3) presence of any number of juvenile polyps and family history of JPS [4]. In general, almost all of the polyps from patients with JPS are typical pedunculated juvenile polyps. A review of 366 polyps obtained from patients with JPS revealed that 90.5% were pedunculated and 9.5% were sessile [16]. The authors found that most of the polyps (363, 99.2%) had benign histology (inflammatory changes) and three (0.8%) involved focal adenomatous changes [16]. In 1982, Perzin KH reported the first case of adenomatous changes found within a gastrointestinal hamartomatous polyp [17]. About 7 and 14% of the 88 polyps in patients with JPS had been reported to be adenoma and low grade dysplasia, respectively [18]. Other authors described a patient with JPS with an *SMAD4* mutation who displayed unexplained adenomatous polyposis [19]. Another report described a patient with *SMAD4* mutations who displayed 20–99 adenomatous polyps and no juvenile polyps [20]. Hereditary mixed polyposis syndrome (HMPS) is a disease characterized by mixed hyperplastic, adenomatous, and atypical juvenile polyps in the colon and rectum [21]. *BMPR1A* gene mutation is the reported cause of HMPS [22, 23]. The collective observations indicate that patients with JPS may sometimes have concomitant adenomatous polyps, which would lead to the misdiagnosis of JPS as FAP in clinical practice. In our study, both of the two examined polyps displayed dysplasia. It is difficult for a pathologist to differentiate the juvenile polyps with dysplasia from adenoma. So, juvenile polyp with dysplasia can often be diagnosed as adenoma. JPS patients with dysplasia, especially those with concomitant colorectal adenocarcinoma, might be easily misdiagnosed as FAP. If dysplasia is found in a local area of the background of typical juvenile polyp, the pathological diagnosis of juvenile polyp can be easily made. But if only few polyps were examined, they might be misdiagnosed as adenomas, and the patient might be misdiagnosed with FAP. The presence of multiple pedunculated polyps and pathological examination of more polyps could help ensure an accurate diagnosis. In addition, the endoscopic features of polyps could also help us to differentiate FAP from JPS. In general, most polyps in FAP are small sessile polyps, while the juvenile polyps are usually pedunculated with irregular surface.

Germline mutations in *SMAD4* or *BMPR1A* have been reported in 50–60% of patients with JPS [4, 7–9]. JPS belongs to the group of hamartomatous polyposis, which also includes Peutz-Jeghers syndrome (caused by germline *STK11* mutation) and Cowden syndrome (caused by germline *PTEN* mutation). Their manifestations differ only slightly and only gene mutation analysis can verify the clinical diagnosis [24]. It is sometimes difficult to differentiate those three types of hamartomatous polyposes without genetic mutation results. For these reasons, germline gene mutation is very important to make the accurate diagnosis of JPS.

Most polyps in patients with JPS are benign hamartomatous polyps with no dysplasia. A review of 767 colorectal JPS polyps demonstrated that 8.5% of the polyps contained mild to moderate dysplasia, and only 0.3% had severe dysplasia or cancer found in carpeting polyps [25]. To reduce the malignant risk, all polyps that are found in the gastrointestinal tract of patients with JPS should be removed endoscopically. Patients with mild polyposis can be managed by frequent endoscopic resection. Prophylactic gastrectomy and colectomy should be considered for patients with severe, large polyps, because of the difficulty of endoscopic treatment and the high recurrence rate of polyps [3, 6, 26]. There is no consensus on the indications for colectomy in JPS. Current indications include excessive polyp burden that is too difficult or perilous to manage endoscopically, persistent blood loss resulting in persistent anemia or hypoalbuminemia [27], juvenile polyps with severe dysplasia, malignant polyps, and a strong family history of colorectal cancer [24]. Surgical options include segmental colectomy, subtotal colectomy, total colectomy (TC) with ileal rectal anastomosis (IRA), and TPC + IPAA [3]. A publication from the Cleveland Clinic reported that, among 35 patients with JPS, 8 had TPC and IPAA, 13 had total colectomy, and 2 had segmental colectomy [28]. A review of 171 patients with JPS without malignant tumors in Japan showed that 20.2, 7.1, and 6.0% were treated by segmental colectomy, TC + IRA, and TPC + IPAA, respectively [29]. A report of 44 JPS patients treated at St Mark’s Hospital demonstrated that only one patient received TC + IRA and one received TPC + IPAA [25]. The extent of colorectal resection can be influenced by the extent of rectal polyposis and by the existence and sites of locally advanced or endoscopically unmanageable colorectal cancer. The long-term oncological effects of the different types of surgery remain unclear. The most suitable timing for prophylactic colectomy also remains unknown [29]. Presently, the father was treated by TPC + IPAA becaused he was misdiagnosed as FAP. The son was treated by subtotal colectomy and endoscopic resection of polyps in the rectum and sigmoid colon. For the son, all identified polyps were removed completely, and his urinary, fecal, and sexual functions were preserved. So, it is a suitable treatment for him.

For patients with JPS, continued surveillance is required regardless of the type of surgery performed, since polyps may develop in the rectum or ileal pouch [3]. EGD and total colonoscopy should begin at 15 years of age or earlier if symptoms develop, and all identified polyps should be removed [3, 4, 16]. Screening endoscopy should be repeated annually if polyps are found and every 2 to 3 years if no polyps are identified [5, 26]. Although small bowel polyps and duodenal polyps have also been found in patients with JPS [30], small bowel disease is not a significant clinical problem in JPS and surveillance should not be performed [6, 25]. In patients with JPS with *SMAD4* mutation, screening for signs of HHT should be considered, which include chest radiography for arteriovenous malformations, magnetic resonance imaging of the brain, and liver ultrasonography [5].

In conclusion, it is difficult to differentiate juvenile polyps with dysplasia from adenomatous polyps, which could explain why juvenile polyps have been reported to have adenomatous changes in patients with JPS. Therefore, patients with JPS, especially those with concomitant dysplasia and adenocarcinoma, might be easily misdiagnosed as FAP in clinical practice. Patients with multiple pedunculated polyps should be suspected for JPS. Sampling more polyps for pathological examination and germline gene mutation tests might help aid an accurate diagnosis. The differential diagnosis of JPS versus FAP, should be based on comprehensive evaluation of clinical presentation, endoscopic appearance and genetic investigations (Table 1); and not merely based on the presence or absence of adenomatous polyp (dysplasia in the polyps).

**Table 1:** Differences in clinicopathological characteristics and treatment between JPS and FAP

Parameters	JPS	FAP
Clinical presentation	Multiple juvenile polyps throughout the gastrointestinal tract. The lifetime cancer risk is 10–50%.	Early onset of hundreds to thousands of polyps throughout the colorectum. The lifetime cancer risk is 100% by age 50.
Endoscopic appearance	The polyps vary in size, shape and number. Typical: pedunculated, strawberry shape, 5~100 polyps.	Classical: 100-thousands polyps, Attenuated: < 100 polyps.
Pathological findings	Hamartomatous polyps.	Adenomatous polyps.
Extracolonic lesions	Gastric cancer, small intestine polyp, pancreas cancer, hereditary hemorrhagic telangiectasia (HHT)	CHRPE, epidermoid cysts, osteoma, desmoid tumor, hepatoblastoma, supernumerary teeth, thyroid cancer, brain tumor
Genetic investigations	Autosomal dominant, about 50–60% had germline *SMAD4* or *BMPR1A* mutations	Autosomal dominant, about 80% had germline *APC* mutation.
Treatment	Most could be treated by polypectomy. Colectomy is rarely needed.	Most require TC + IRA or TPC + IPAA.

*CHRPE* Congenital hypertrophy of the retinal pigmented epithelium

## Supplementary information

**Additional file 1 Supplemental Table 1**. The 139-gene panel used in the genetic test

## Data Availability

The datasets used and analysed during the current study are available from the corresponding author on reasonable request.

## Abbreviations

BMPR1A: Bone morphogenetic protein receptor type 1A
EGD: Esophagogastroduodenoscopy
FAP: Familial adenomatous polyposis
HHT: Hereditary hemorrhagic telangiectasia
HMPS: Hereditary mixed polyposis syndrome
IPAA: Ileal pouch anal anastomosis
IRA: Ileal rectal anastomosis
JPS: Juvenile polyposis syndrome
PJS: Peutz-Jeghers syndrome
SMAD4: SMAD family member 4
TC: Total colectomy
TPC: Total proctocolectomy
