# Supplementary material for: Juvenile polyposis syndrome might be misdiagnosed as familial adenomatous polyposis: a case report and literature review
Source: BMC Gastroenterol. 2020 Jun 1;20:167. doi: 10.1186/s12876-020-01238-7 (PMC7268223; doi:10.1186/s12876-020-01238-7)
Supplement: Supplementary file 1 — Additional file 1 Supplemental Table 1. The 139-gene panel used in the genetic test [file 12876_2020_1238_MOESM1_ESM.docx]

**Supplemental Table 1**. The 139-gene panel used in the genetic test

| *AIP* | *CBL* | *EGFR* | *FANCD2* | *GPC3* | *MLH3* | *PDGFRA* | *RAD50* | *SDHC* | *TP53* |
| --- | --- | --- | --- | --- | --- | --- | --- | --- | --- |
| *ALK* | *CDC73* | *ELANE* | *FANCE* | *GREM1* | *MRE11A* | *PHOX2B* | *RAD51B* | *SDHD* | *TSC1* |
| *APC* | *CDH1* | *EPCAM* | *FANCF* | *HMBS* | *MSH2* | *PMS1* | *RAD51C* | *SLX4* | *TSC2* |
| *ATM* | *CDK4* | *ERCC1* | *FANCG* | *HNF1A* | *MSH6* | *PMS2* | *RAD51D* | *SMAD4* | *UROD* |
| *ATR* | *CDKN1B* | *ERCC2* | *FANCI* | *HOXB13* | *MTAP* | *POLD1* | *RB1* | *SMARCA4* | *USHBP1* |
| *AXIN2* | *CDKN1C* | *ERCC3* | *FANCL* | *HRAS* | *MTUS1* | *POLE* | *RECQL* | *SMARCB1* | *VEGFA* |
| *BAP1* | *CDKN2A* | *ERCC4* | *FANCM* | *KIT* | *MUTYH* | *POLH* | *RECQL4* | *SMARCE1* | *VHL* |
| *BARD1* | *CEBPA* | *ERCC5* | *FAS* | *LASP1* | *NBN* | *PPM1D* | *RET* | *SOS1* | *WRN* |
| *BLM* | *CHEK1* | *EXT1* | *FH* | *MAX* | *NF1* | *PRKAR1A* | *RHBDF2* | *STAT3* | *WT1* |
| *BMPR1A* | *CHEK2* | *EXT2* | *FLCN* | *MC1R* | *NF2* | *PRSS1* | *RUNX1* | *STK11* | *XPA* |
| *BRCA1* | *CYLD* | *EZH2* | *GALNT12* | *MEN1* | *NSD1* | *PTCH1* | *SBDS* | *SUFU* | *XPC* |
| *BRCA2* | *DDB2* | *FANCA* | *GATA2* | *MET* | *NTRK1* | *PTCH2* | *SDHA* | *TERT* | *XRCC2* |
| *BRIP1* | *DICER1* | *FANCB* | *GEN1* | *MITF* | *PALB2* | *PTEN* | *SDHAF2* | *TGFBR1* | *ZMAT3* |
| *BUB1B* | *DIS3L2* | *FANCC* | *GJB2* | *MLH1* | *PALLD* | *PTPN11* | *SDHB* | *TMEM127* |  |
